# Supplementary material for: Biophysics-based protein language models for protein engineering
Source: Nat Methods. 2025 Sep 11;22(9):1868–79. doi: 10.1038/s41592-025-02776-2 (PMC12446067; doi:10.1038/s41592-025-02776-2)
Supplement: Supplementary file 2 — Reporting Summary [file 41592_2025_2776_MOESM2_ESM.pdf]

nature portfolio

Corresponding author(s): Anthony Gitter and Philip Romero

Last updated by author(s): Apr 22, 2025

Reporting Summary

Nature Portfolio wishes to improve the reproducibility of the work that we publish. This form provides structure for consistency and transparency in reporting. For further information on Nature Portfolio policies, see our Editorial Policies and the Editorial Policy Checklist.

Statistics

For all statistical analyses, confirm that the following items are present in the figure legend, table legend, main text, or Methods section.

n/a	Confirmed	
☐	☒	The exact sample size (*n*) for each experimental group/condition, given as a discrete number and unit of measurement
☐	☒	A statement on whether measurements were taken from distinct samples or whether the same sample was measured repeatedly
☐	☒	The statistical test(s) used AND whether they are one- or two-sided<br>*Only common tests should be described solely by name; describe more complex techniques in the Methods section.*
☒	☐	A description of all covariates tested
☒	☐	A description of any assumptions or corrections, such as tests of normality and adjustment for multiple comparisons
☐	☒	A full description of the statistical parameters including central tendency (e.g. means) or other basic estimates (e.g. regression coefficient) AND variation (e.g. standard deviation) or associated estimates of uncertainty (e.g. confidence intervals)
☐	☒	For null hypothesis testing, the test statistic (e.g. *F*, *t*, *r*) with confidence intervals, effect sizes, degrees of freedom and *P* value noted<br>*Give P values as exact values whenever suitable.*
☒	☐	For Bayesian analysis, information on the choice of priors and Markov chain Monte Carlo settings
☒	☐	For hierarchical and complex designs, identification of the appropriate level for tests and full reporting of outcomes
☒	☐	Estimates of effect sizes (e.g. Cohen's *d*, Pearson's *r*), indicating how they were calculated

Our web collection on statistics for biologists contains articles on many of the points above.

Software and code

Policy information about availability of computer code

Data collection	The experimental data was collected on a Tecan plate reader using the Magellan software.
Data analysis	For running molecular simulations<br>Rosetta (3.13) - Modeling and analysis of protein structures<br>Python (3.9) - Primary programming language for all analysis<br>NumPy (1.19.5) and Pandas (1.2.1) - Numerical computations and data manipulation<br>Matplotlib (3.3.4) and Seaborn (0.11.1) - Plotting and data visualization<br>ShortUUID (1.0.1) - Assigning unique identifiers to computational runs<br>Biopython (1.78) - Manipulating protein structures<br>JupyterLab (3.0.12) - Interactive environment for method development and data analysis<br>SQLAlchemy (1.4.39) - SQL data management<br>OpenSSL (3.1.1) - Encrypting software<br><br>Custom code for running molecular simulations can be found at https://github.com/gitter-lab/metl-sim<br><br>For training and evaluating neural networks<br>Python (3.9.13) - Primary programming language for all analysis<br>PyTorch (1.12.1), PyTorch Lightning (1.7.7), TorchMetrics (0.10.2), and TorchExtractor (0.3.0) - Training and evaluating neural networks<br>NumPy (1.23.4), Pandas (2.0.3), SciPy (1.9.3) - Numerical computations and data manipulation<br>Scikit-learn (1.1.3) - Baseline linear regression models and utility functions<br>NetworkX (2.6.3) - Processing protein structures as graphs

Matplotlib (3.7.2), Seaborn (0.12.2), and AdjustText (0.8) - Plotting and visualizing data
UMAP-Learn (0.5.5) - Data dimensionality reduction and visualization
Jupyter (1.0.0), JupyterLab (4.1.2), and IPyWidgets (8.1.2) - Interactive environment for method development and data analysis
WandB (0.13.5) - Weights and biases experiment tracking
ShortUUID (1.0.1) - Assigning unique identifiers to computational runs
BioPandas (0.2.7) and Biopython (1.83) - Manipulating protein structures
SQLAlchemy (1.4.43), PyTables (3.7.0), and ConnectorX (0.3.2) - SQL data management
Fair-ESM (2.0.0) - Benchmarking ESM
MMseqs2 (15.6f452) and Foldseek (9.427df8a) - Sequence- and structure-based clustering
SparkControl (2.3) - Collect data from Tecan plate reader

Custom code for training and evaluating neural networks can be found at https://github.com/gitter-lab/metl and https://github.com/gitter-lab/metl-pretrained and https://github.com/gitter-lab/metl-pub

In addition to these software libraries, we note that numerous software dependencies were automatically installed by our package managers (Conda, Mamba, and pip — multiple versions), as part of the environment set up. These dependencies are important to the functionality of the primary software packages listed above.

For manuscripts utilizing custom algorithms or software that are central to the research but not yet described in published literature, software must be made available to editors and reviewers. We strongly encourage code deposition in a community repository (e.g. GitHub). See the Nature Portfolio guidelines for submitting code & software for further information.

Data

Policy information about availability of data

All manuscripts must include a data availability statement. This statement should provide the following information, where applicable:

- Accession codes, unique identifiers, or web links for publicly available datasets
- A description of any restrictions on data availability
- For clinical datasets or third party data, please ensure that the statement adheres to our policy

Pretrained METL models are available at doi:10.5281/zenodo.11051644. Rosetta simulation datasets are available at doi:10.5281/zenodo.10967412. Additional data is available in the GitHub repository https://github.com/gitter-lab/metl- pub, which is archived at doi:10.5281/zenodo.10819536. The PDB structure identifiers used to train METL-Global are listed in Supplementary Table 2. The PDB and AlphaFold DB structure identifiers used for METL-Local are listed in Supplementary Table 7. Experimental datasets used for model evaluation are listed in Supplementary Table 8 with references and corresponding filenames.

Human research participants

Policy information about studies involving human research participants and Sex and Gender in Research.

Reporting on sex and gender	n/a
Population characteristics	n/a
Recruitment	n/a
Ethics oversight	n/a

Note that full information on the approval of the study protocol must also be provided in the manuscript.

Field-specific reporting

Please select the one below that is the best fit for your research. If you are not sure, read the appropriate sections before making your selection.

☒ Life sciences ☐ Behavioural & social sciences ☐ Ecological, evolutionary & environmental sciences

For a reference copy of the document with all sections, see nature.com/documents/nr-reporting-summary-flat.pdf

Life sciences study design

All studies must disclose on these points even when the disclosure is negative.

Sample size	We selected 20 GFP variants for experimental testing to strike a balance between practical constraints—such as limits on DNA synthesis and screening capacity—and the need to evaluate a diverse range of sequences. This set size also allowed us to test a variety of design scenarios. For computational sampling, we chose sample sizes proportionate to the training set sizes. In cases with larger training sets, we observed low variance in outcomes, suggesting that our chosen sample sizes were sufficient.
Data exclusions	No data was excluded

Replication	The experimental findings were confirmed through three experimental replicates of independent protein expression samples.
Randomization	Randomization and blinding were not applicable in this study because all GFP variants were synthesized and tested under identical conditions in a single, controlled experiment. Since all samples were processed uniformly and measured at the same time, there was no risk of bias that randomization or blinding would typically mitigate.
Blinding	Randomization and blinding were not applicable in this study because all GFP variants were synthesized and tested under identical conditions in a single, controlled experiment. Since all samples were processed uniformly and measured at the same time, there was no risk of bias that randomization or blinding would typically mitigate.

Reporting for specific materials, systems and methods

We require information from authors about some types of materials, experimental systems and methods used in many studies. Here, indicate whether each material, system or method listed is relevant to your study. If you are not sure if a list item applies to your research, read the appropriate section before selecting a response.

Materials & experimental systems

n/a	Involved in the study
☒	☐ Antibodies
☒	☐ Eukaryotic cell lines
☒	☐ Palaeontology and archaeology
☒	☐ Animals and other organisms
☒	☐ Clinical data
☒	☐ Dual use research of concern

Methods

n/a	Involved in the study
☒	☐ ChIP-seq
☒	☐ Flow cytometry
☒	☐ MRI-based neuroimaging
